# Exploring the potential of Eu^3+^ and Mn^4+^ activated LaAlO_3_ phosphors as red and far-red emitters for horticulture lighting

**DOI:** 10.1039/d3ra03241h

**Published:** 2023-10-26

**Authors:** S. K. Jakka, M. M. P. Silva, M. J. Soares, K. Pavani

**Affiliations:** a I3N & Physics Department, University of Aveiro Aveiro 3810-193 Portugal suresh@ua.pt jskphysics@yahoo.co.in pavani@ua.pt pavani_physics@yahoo.co.in

## Abstract

The development of efficient red and far-red emitters, for efficient plant absorption in the Photosynthetically Active Radiation (PAR) region, holds significance in contemporary plant growth control. This study focuses on the synthesis and characterization of LaAlO_3_ as a host material, doped with Eu^3+^ and Mn^4+^ ions, using a solid-state reaction method. The investigation encompasses the creation and analysis of both single-doped and co-doped samples, employing techniques including X-ray diffraction (XRD), scanning electron microscopy (SEM), and photoluminescence (PL) spectroscopy. XRD analysis consistently confirmed the perovskite-like structure of all samples, devoid of detectable impurities or major structural changes due to doping. SEM images revealed a uniform distribution of regularly shaped particles for the co-doped sample. The PL spectroscopy showed that the doping led to strong photoluminescence, with the co-doped sample exhibiting the intensity of each of the ions independently neither exhibiting quenching nor energy transfer mechanisms. The excitation spectrum of Eu^3+^ exhibited a broad charge transfer band at approximately 328 nm, coupled with characteristic f–f excitation bands. On the other hand, the Mn^4+^ ion's excitation spectrum featured transitions from ground state (4A_2g_) electrons excited to higher excited states (4T_1g_, 2T_2g_, and 4T_2g_) centered at 350 nm and within the region 250–550 nm. The co-doped sample was excited at a common excitation wavelength of 460 nm and underwent an in-depth examination of its photoluminescent properties, including decay curves analysis and time dependence also. The results from this study suggest that the synthesized phosphor materials exhibit substantial potential for diverse applications, including but not limited to solid-state lighting for efficient plant growth.

## Abstract 2

Contour of emission intensity variation in LAO:Eu/Mn phosphor with change in excitation wavelength.

## Graphical abstract

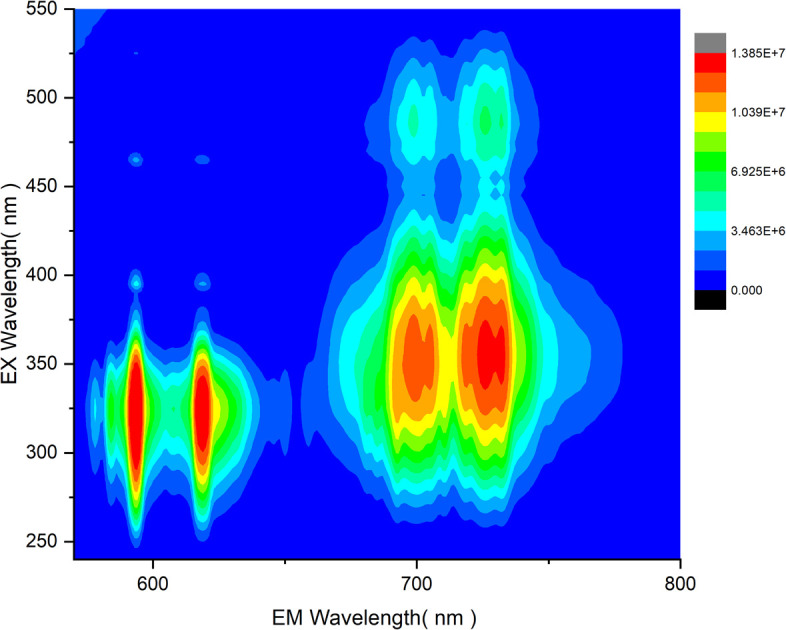

## 1. Introduction

Luminescence is a phenomenon in which a material emits radiation in the form of light, following exposure to a stimulus, such as chemical, electrical, or mechanical actions.^1^ The phenomenon can be categorized into various types based on the nature of the stimulus. One of these categories is photoluminescence, which occurs when a material emits radiation as a result of absorption of photons with higher energy than the radiation it emits during the absorption process.^2^ Materials that exhibit luminescence in the form of powders are commonly known as phosphors. Phosphors typically consist of a host lattice, which makes up the bulk of the material, and intentionally doped impurities (ions) known as dopants existing in relatively small quantities.^3^ It is these dopants that play a key role in determining the luminescent properties of the material, as they are excited and subsequently emit the radiation. The phosphor materials have enormous applications not only in the fields of lighting but also in the multi-disciplinary areas, including security, medical imaging, display technology, energy and environmental monitoring *etc.*

Phosphor materials often contain rare-earth (RE) elements,^3^ also known as lanthanides and in some cases transition metals (TM) also.^4^ One of the RE^3+^ ions that consists of partially filled f-orbitals and is highly efficient in the red region is Europium (Eu^3+^).^5^ On the other hand, TM ions are a group of elements characterized by their partially filled d-orbitals, or the ability to form such orbitals. One such TM ion is Manganese (Mn) in its 4+ state and is known for its efficient far-red emission.^6–8^ For transition metals, the crystal field splitting of the d-orbitals can result in the appearance of a broad emission band due to the transitions between the split energy levels. These are known as d–d transitions.^9^ In contrast, rare-earth ions have narrow emission lines due to transitions between f-orbitals, which are more shielded from the crystal field effect and therefore, less split. Additionally, rare-earth ions can exhibit strong magnetic dipole transitions, which are not observed in transition metal ions.^10^ Overall, the crystal environment plays a crucial role in determining the luminescent properties of phosphors and is an important consideration in the design of new materials for various applications.

Plants possess a variety of photoreceptors that respond to different light wavelengths. In addition to phytochrome, there are also blue-light photoreceptors called cryptochromes and phototropins, along with UV-B photoreceptors called UVR8.^11^ These photoreceptors are proteins capable of absorbing light and initiating specific cellular responses, such as changes in gene expression or enzyme activity.

Plants have evolved complex mechanisms for detecting and reacting to varying light wavelengths, enabling them to adapt to their surrounding environments and optimize their growth and development. The range of light wavelengths from 400–700 nm, referred to as Photosynthetically Active Radiation (PAR), is particularly vital for photosynthesis. Although other wavelengths of radiation beyond the PAR range can also contribute to plant growth, the energy in the PAR range is the most efficiently absorbed and used by plant cells.^12^

**Fig. 1:**
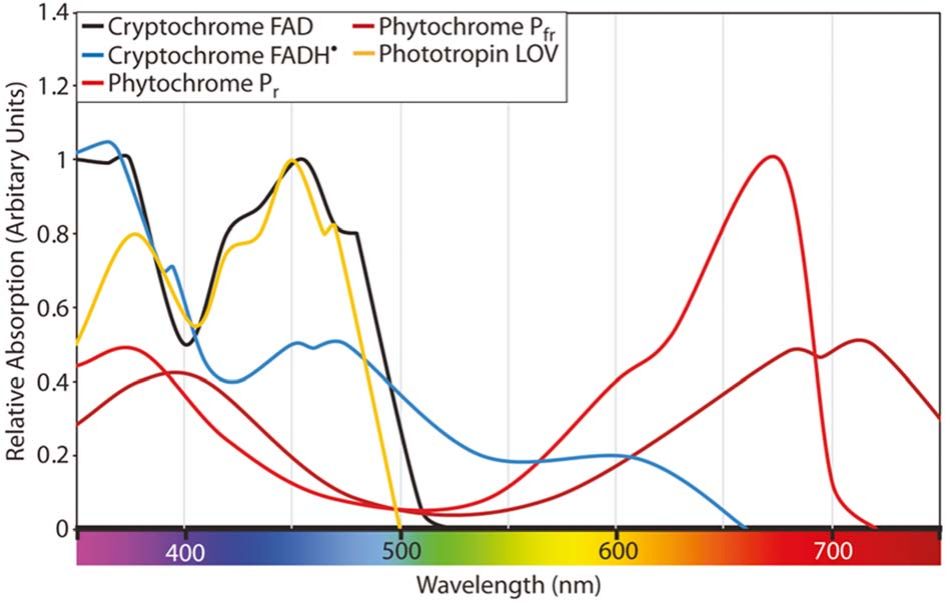
Absorption spectra for phytochrome, cryptochrome, and phototropin of plants as presented by Martin W. Battle *et al.*^13^ redrawn from different sources.^14,15^

Fig. 1 illustrates the absorption spectra for phytochrome, cryptochrome, and phototropin of plants, as originally presented by Martin W. Battle *et al.*^13^ and redrawn from various other sources.^14,15^ Within the PAR range, different photoreceptors exhibit have varying degrees of relative absorption in different regions. Photochrome, a plant photoreceptor protein, exists in two forms: phytochrome P_fr_ (far-red absorbing form) and P_r_ (red absorbing form). They play pivotal roles in numerous critical processes in plant growth and development. Phytochrome P_fr_ plays a crucial role in regulating seed germination, plant growth, and flowering.^16^ It triggers the opening of stomata in response to light, enabling the exchange of gases required for photosynthesis. It also regulates gene expression, leading to the production of specific proteins that are necessary for plant growth and development. Phytochrome P_r_ is essential for detection of light quality and quantity. It is responsible for detecting the presence of red light, which signals the plant to commence photosynthesis. It also contributes to the regulation of plant growth and development by controlling the production of specific proteins essential for these processes.

When it comes to selecting phosphor materials for LED manufacturing, different strategies must be developed for specific purposes. In the context of horticulture, the use of red and far-red emitting LEDs is of paramount important for effectively illuminating plants and promoting growth and germination. To explore various dopant ions that emit red and far-red, a combination of Eu^3+^ and Mn^4+^ ions has been identified as a promising approach, as it can yield highly efficient light emission to the P_fr_ and P_r_ regions. Lanthanum aluminate (LAO) is a kind of inorganic compound with the chemical formula LaAlO_3_. It is an optically transparent ceramic oxide with a distorted perovskite structure. Crystalline LAO possesses a relatively high relative dielectric constant of approximately 25. The crystal structure of LAO is rhombohedral distorted perovskite with a pseudocubic lattice parameter of 3.787 Å at room temperature.^17^ Importantly, in this inorganic compound it is both feasible and flexible to introduce either RE or TM ions in the La or Al sites, respectively. In this particular study, we have chosen to dope Eu in place of La ions and Mn in place of Al ions. This strategic doping is designed to enable efficient emission of red and far-red light in the development of LEDs tailored for horticulture applications.

## 2. Experimental section

The ceramic powder samples were prepared using a high temperature solid state reaction method. Metal oxide precursors La_2_O_3_, Al_2_O_3_, Eu_2_O_3_ and MnO_2_ were weighted based on the stoichiometric ratios in the present formula:La_2(1−*x*)_O_3_ + Al_2(1−*y*)_O_3_ + *x*Eu_2_O_3_ + 2*y*MnO_2_ → 2La_(1−*x*)_Al_(1−*y*)_O_3_:Eu_2*x*_Mn_2*y*_

Using this formula, four samples were made (*x* = 0, *y* = 0; *x* = 5, *y* = 0; *x* = 0, *y* = 0.5 and *x* = 5, *y* = 0.5 mol%). After weighting with appropriate molar ratios, the materials were ground using an agate mortar with a small amount of ethanol to ensure homogeneity. The mixtures were then placed into alumina crucibles and sintered at 1500 °C for 5 h in a muffle furnace. Subsequently they were cooled to room temperature and molded into pellets of 7 mm diameter for further qualitative and quantitative analysis.

Crystal structure and phase identification were carried out using powder X-ray diffraction (XRD) using a Philips X'pert MPD diffractometer (Bragg-Brentano geometry), operating with Cu Kα radiation (*λ* = 1.5405980 Å) at 40 kV and 50 mA, scanning the samples in the 2*θ* range of 20 to 70°. The morphology of the prepared phosphor materials were assessed using scanning electron microscope (SEM) TESCAN VEGA3, which was used for imaging and evaluating the microstructural characteristics of LaAlO_3_ host material with the dopants Eu^3+^ and Mn^4+^ after the preparation. SEM images were acquired with a voltage of 30 kV and a magnification of 5000×.

Photoluminescence (PL) emission and excitation spectra were obtained using a Horiba Jobin Yvon fluorolog-3 spectrofluorometer equipped with a Xe lamp with excitation ranging from 240 to 650 nm. The sample's emission covered a wavelength range from 250 to 850 nm. The photons emitted by the sample were then detected by an orthogonally placed photo multiplier tube, which detected the intensity of the radiation. As excitation and emission monochromators could be controlled, it is possible to record both excitation and emission respectively. The PL decay curves were recorded using Xe flash lamp.

## 3. Results and discussion

### 3.1. Structural characterisation

**Fig. 2:**
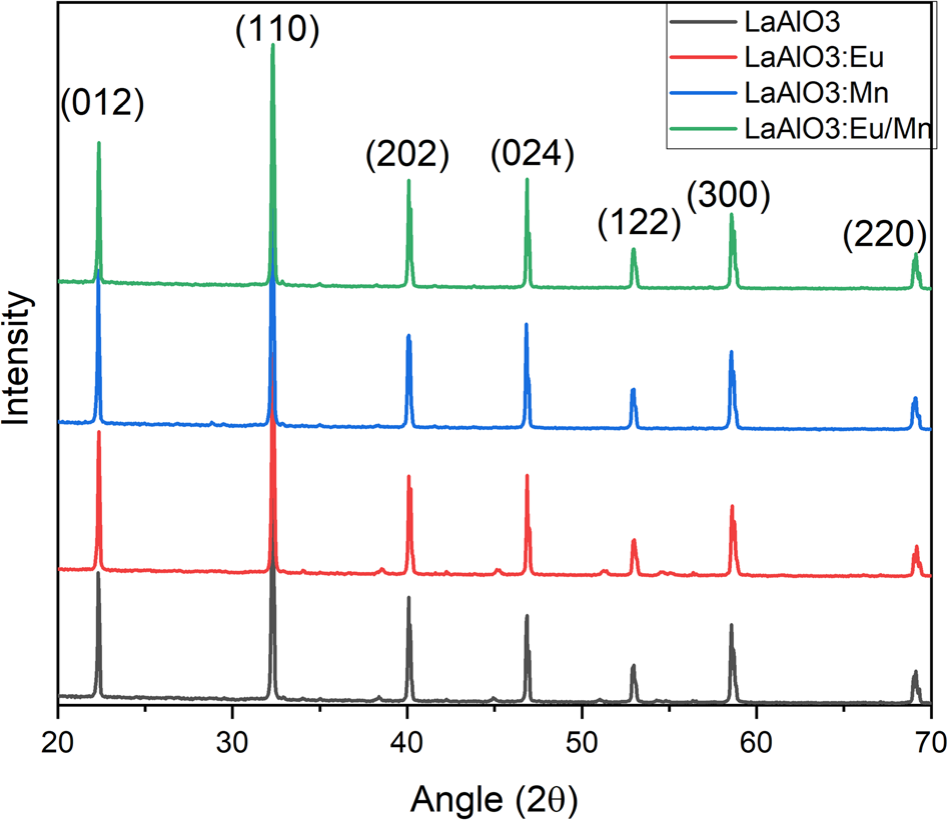
X-ray diffraction of LAO host before and after doping Eu^3+^ and Mn^4+^ ions.

The X-ray powder diffraction patterns for all the samples, whether in pure host form or with the dopants, exhibited striking similarities and revealed a perovskite-like structure as presented in Fig. 2. The (*hkl*) miller indices of the XRD pattern were also included in the illustration. The identified crystalline peaks were well matched to the LaAlO_3_ crystal structure of perovskite type with JCPDS file (PDF card number 031-0022). The nearly identical XRD patterns suggest that the dopants may have replaced and occupied the host atoms. Specifically, Eu^3+^ took the place of La^3+^ ions, while Mn^4+^ occupied the position of Al^3+^ ions. Due to the nearly similar atomic sizes of the dopants and the replaced ions in the host lattice, no major changes in XRD pattern have been observed as well as no detectable peaks from possible impurities are found. This indicates that the dopants enter the host lattice in a homogeneous way and preserve the crystallinity of the structure.^18^ It should be noted that with increase in dopant concentration, the material's crystal structure would undergo more pronounced changes, potentially leading to the formation of sublattices, that could have a detrimental impact on the desired structural homogeneity.

**Fig. 3:**
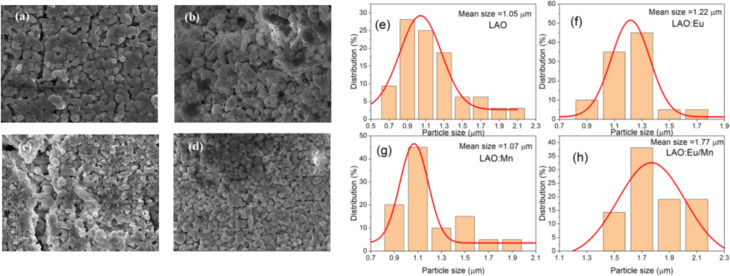
(a–d) Morphological SEM images of the prepared (a) LAO, (b) LAO:Eu, (c) LAO:Mn and (d) LAO:Eu/Mn and (e–h) particle size distribution based on the SEM images, respectively.

The SEM images and particle size distribution are illustrated in Fig. 3. They provide insight into the characteristics of the prepared samples. From the SEM images in Fig. 3(a–d) it could be inferred that the particles are distinguishable and roughly uniform. Each particle appears to have a diameter of approximately 1 μm for host material, and in the case of doped phosphors slight increase of particle size is observed. The mean particle size increased to 1.07 μm for Mn^4+^ doped LAO (Fig. 3(f)) and 1.22 μm for Eu^3+^ doped LAO (Fig. 3(g)) phosphors respectively. Eu^3+^ and Mn^4+^ co-doped LAO phosphor sample is found to have the maximum mean particle size with 1.77 μm (Fig. 3(h)). The relatively larger size of the particles can likely be attributed to the high temperature synthesis process employed in the solid-state reaction method. It is worth noting that employing alternatively chemical preparation methods namely coprecipitation, sol–gel, or hydrothermal methods, *etc.*, could have substantial effect with smaller particle size as well as more uniform distribution.

### 3.2. Luminescence characterisation

**Fig. 4:**
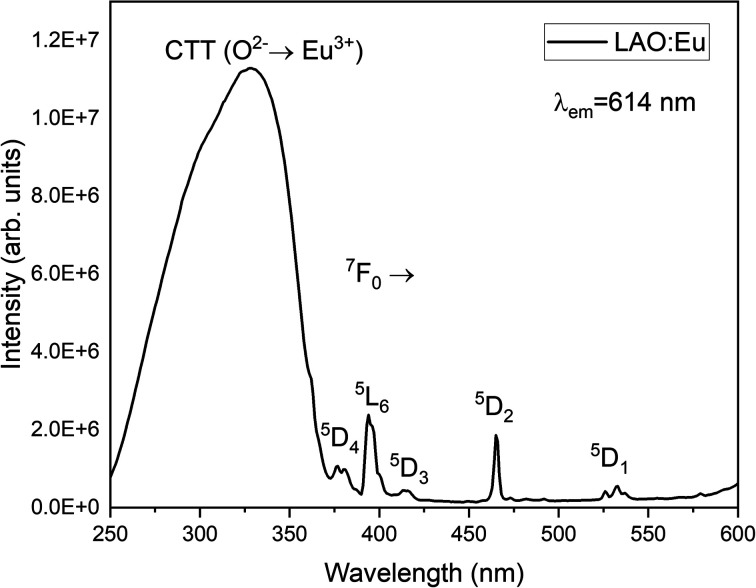
Excitation spectrum of LAO:Eu monitoring emission at 614 nm radiation.

Fig. 4 illustrates the luminescence excitation spectra of LAO:Eu. This excitation spectrum was recorded by monitoring the emission at 619 nm and reveals a dominant broad band with its peak at approximately 328 nm. This broad band can be attributed to a charge transfer transition (CTT) occurring from O^2−^ to Eu^3+^ ions. This transition signifies a shift from the valence band to the localized Eu^2+^ state, resulting in the creation of europium trapped excitons (EuTE). EuTE is regarded as a Eu^2+^ system, with a hole attracted by the Coulomb potential of negatively charged Eu^2+^ (since Eu^2+^ replaces the La^3+^), bound at an energy state above the valence band.^19,20^ All the other minor excitation peaks in the excitation spectra of LAO:Eu are attributed to the f–f transitions of Eu^3+^ ions in LAO host.

**Fig. 5:**
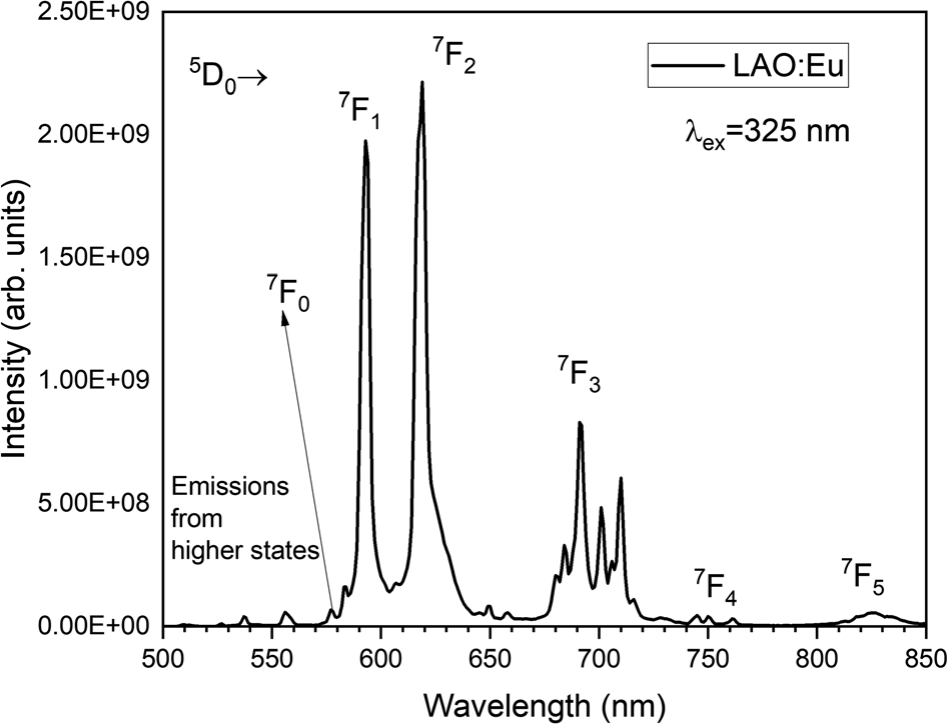
Emission spectrum of LAO:Eu phosphor when excited at 325 nm.

Upon excitation at 325 nm, various emission peaks have been observed. As depicted in Fig. 5, emissions from higher energy states were noted at energies below 560 nm, although they exhibited relatively lower intensities. Conversely, lower emission energies, above 560 nm displayed distinct emission peaks arising from the ^5^D_0_ excited level to different lower lying levels namely ^7^F_0_, ^7^F_1_, ^7^F_2_, ^7^F_3_, ^7^F_4_ and ^7^F_5_ centered around 568, 593, 619, 691, 750 and 824 nm, respectively. Among these bands, the emission peaks around 593, 619 and 691 nm are found to be highly intense and are responsible for the red emission of LAO:Eu phosphor.

**Fig. 6:**
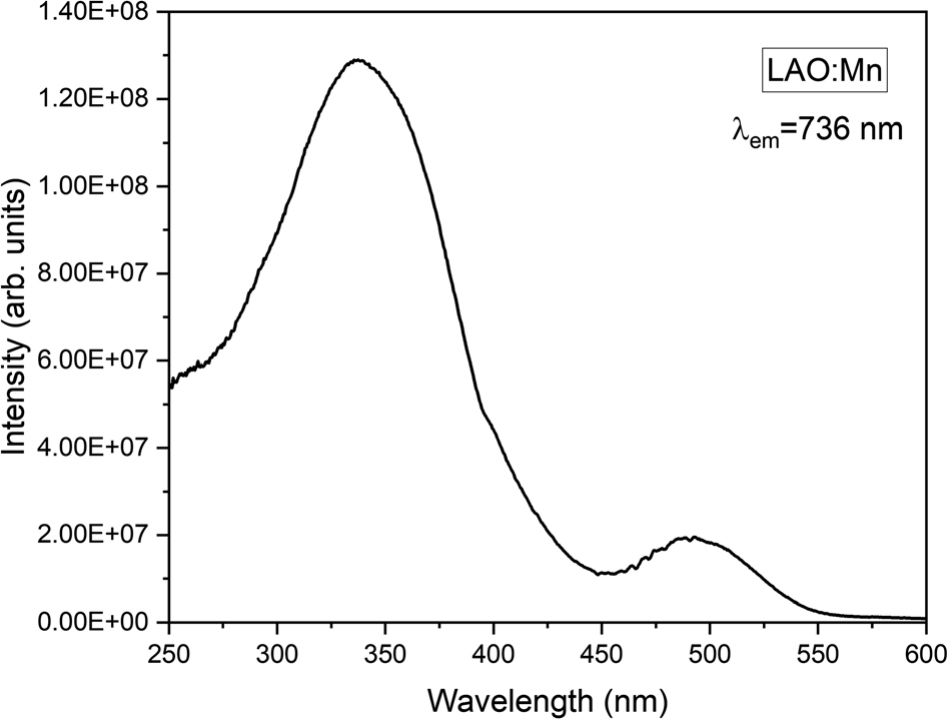
Excitation spectrum of LAO:Mn phosphor monitoring emission at 736 nm radiation.

Mn^4+^ is a transition metal ion characterized by an electronic configuration of 3d^3^. The photoluminescence excitation bands in Mn^4+^ ions occur as the ground state (4A_2g_) electrons of Mn^4+^ ions excite to the higher excited states (4T_1g_, 2T_2g_, and 4T_2g_), while the emission band occurs when the electrons in the excited state (2E_g_) return to the ground state (4A_2g_). The emission of light by Mn^4+^ ions is influenced by the crystal field surrounding the ion. In Fig. 6, the excitation spectrum of the LAO:Mn phosphor is presented, with the emission monitored at 736 nm. A spectrum reveals a convoluted curve representing all the three excitation bands of Mn^4+^ within the range 250–550 nm. These bands arise from a charge transfer transition (CTT) between Mn^4+^–O^2−^, resulting in a transition from the ground state (4A_2g_) to the excited states (4T_1g_, 2T_2g_). Notably when light is absorbed within the range of 220–450 nm, the electrons in Mn^4+^ ions are excited to (4T_2g_) excited state when irradiated between 450 and 550 nm.^21^ Among these broad convoluted excitation bands, the excitation position at 350 nm is found to be the peak maximum.

**Fig. 7:**
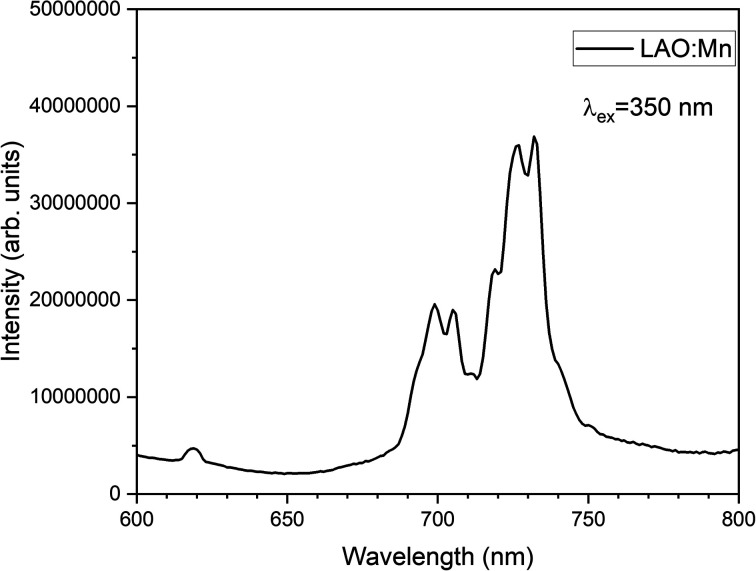
Emission spectrum of LAO:Mn phosphor when excited at 350 nm.

The emission spectrum of LAO:Mn when excited at 350 nm is shown in Fig. 7, in which a highly intense broad band ranging from 690–750 nm corresponding to the transitions of electrons from excited state (2E_g_) returning to the ground state (4A_2g_). The broad band has been split into four sharp peaks *n* the red and far-red range of the spectrum due to the effective crystal field splitting.

**Fig. 8:**
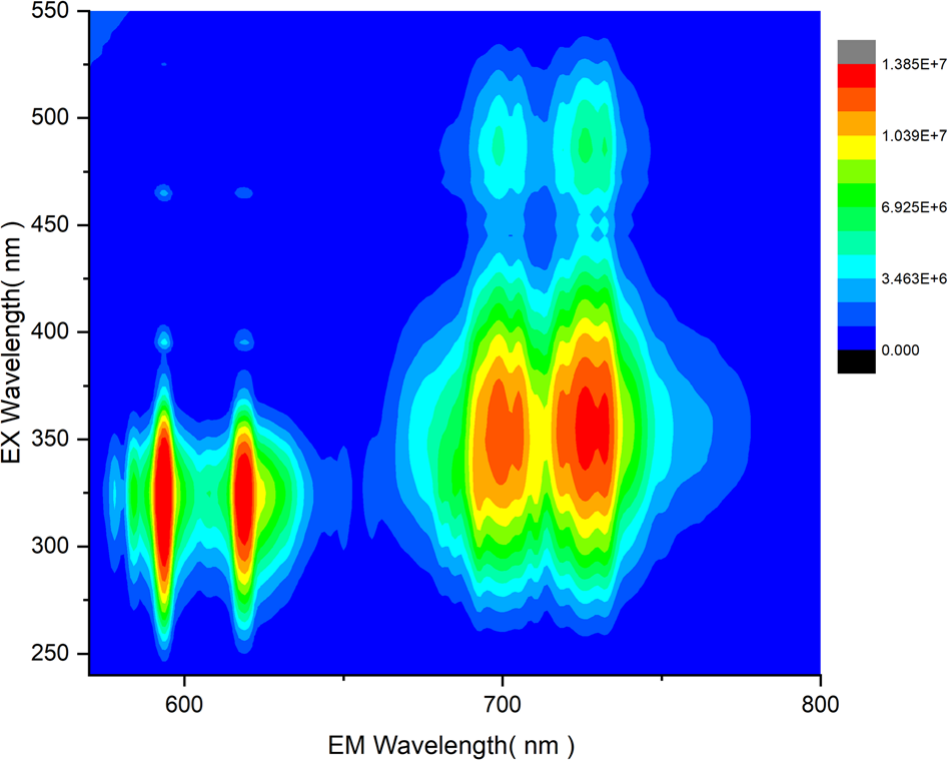
Contour of emission intensity variation in LAO:Eu/Mn phosphor with change in excitation wavelength.

**Fig. 9:**
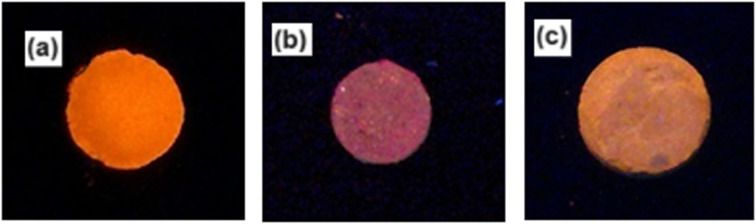
Photographs of (a) LAO:Eu, (b) LAO:Mn and (c) LAO:Eu/Mn phosphor samples under UV (265 nm) excitation.

To further investigate the combination of Mn^4+^ and Eu^3+^ dopants in LAO, the co-dopant sample as mentioned in the experimental section, was subjected to photoluminescent studies. The variation of emission intensity relative to the excitation intensity was assessed and is presented in the contour plot shown in Fig. 8. The three-dimensional contour in Fig. 8 provides clear evidence that an excitation wavelength around 330 nm is well-suited for effective and simultaneous excitation of both the dopant ions, leading to their emission in red and far-red regions. This suggests that an LED emitting between 320 and 350 nm could be an optimized source for the LAO:Eu/Mn. In turn, this phosphor emits in both red and far-red regions simultaneously, enhancing its capacity for effective absorption and greater plant yield. Furthermore, a careful examination of the contour plot also reveal that a source emitting around 460 nm (typical of blue LED) offers nearly equal excitation potential for both the dopant ions. It is essential to note that there is no energy transfer between Eu^3+^ and Mn^4+^ because the excitation of one dopant ion does not overlap with the emission of the other dopant ion (Fig. 4–7) and hence quenching due to co-dopants does not occur. Consequently, a common excitation source can effectively serve both the dopants, ensuring excitation and emission possibilities in the phosphor material. As a reference, in Fig. 9, photographs of the phosphor samples irradiated with UV excitation, offering a visual representation of the emission colors of individually doped phosphor alongside the co-doped LAO material.

**Fig. 10:**
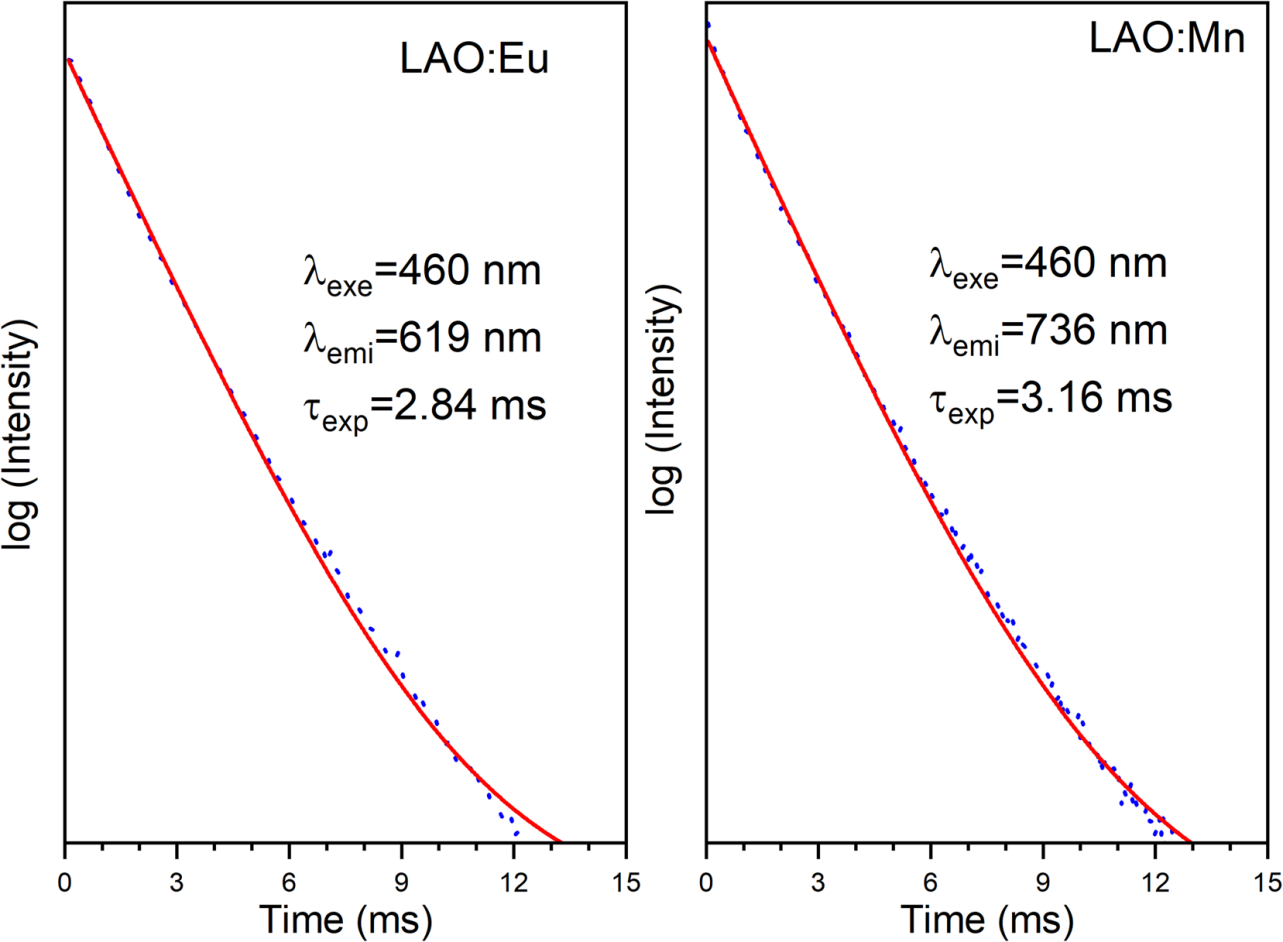
Decay curves of LAO:Eu and LAO:Mn phosphors when excited at 460 nm.

**Fig. 11:**
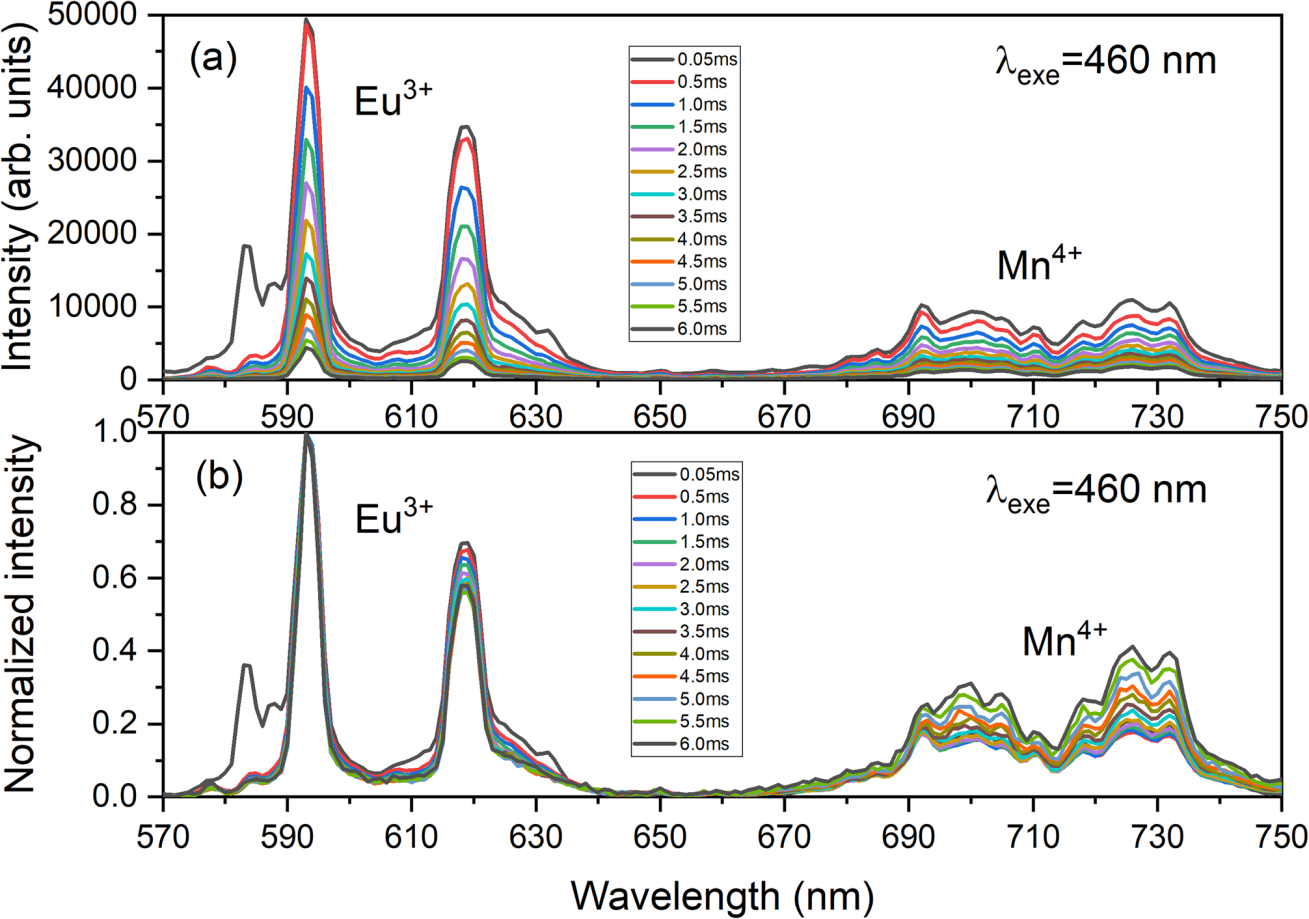
Time dependent (a) as recorded emission spectra and (b) normalized emission spectra of LAO:Eu/Mn phosphor focussing the respective emissions of dopant ions.

Fig. 10 presents the decay curves of both single doped LAO phosphors when excited at the same wavelength of 460 nm. The emission intensities for both the samples were monitored at their maximum wavelengths *i.e.*, for LAO:Eu, it is 619 nm and for LAO:Mn it is at 738 nm, respectively. The decay lifetimes of both the samples was found to 2.84 and 3.16 ms and both were found to be single exponential. From this, it could be inferred that because the decay curves of both the single doped samples are dissimilar, though the likelihood of energy transfer between the two dopant ions is rare, the relative intensities of the emissions from both the dopants might change with time. This could result slight variation in the cumulative emission from both samples over a short period. To validate this inference, the time-dependent emission has been recorded for the LAO:Eu/Mn phosphor exciting at a common excitation wavelength *i.e.*, at 460 nm. Fig. 11 shows the emission spectra of LAO:Eu/Mn excited at 460 nm, recorded at different time interval ranging from 0.05 to 6 ms with an interval of 0.5 ms. It is evident from Fig. 11(a) that the intensity of Eu^3+^ and Mn^4+^ emission bands decreases with increase of time. But the decay time of Mn^4+^ has been observed to be longer compared to that of Eu^3+^ as seen in Fig. 10. This lead to change in relative intensities (Fig. 11(b)) of the emission of Eu^3+^ and Mn^4+^ with an observation that decrease of emission from Mn^4+^ with respect to time is relatively slow and change in the cumulative emission of LAO:Eu/Mn phosphor emission when excited at 460 nm. Also, it is to be observed that the relative intensity of ^5^D_0_ → ^7^F_1_ and ^5^D_0_ → ^7^F_2_ as the transitions are due to magnetic and electric dipole interactions which may have slightly different decay times contributing to the observed changes in emission intensity in Fig. 11(b).

As observed through the changes in the relative intensities in the time-dependent photoluminescence, CIE coordinates were calculated for each of the luminescence spectrum at various time points. It is worth noting that the CIE coordinates remained largely consistent, consistently placing approximately (0.69, 0.31) with minor variations in the fourth decimal place. This suggests that emission colour of the co-doped phosphor material is almost unchanged over time, ensuring a well-controlled emission colour. This feature makes it highly suitable for implementation in pc-LEDs as a combination of red and far-red light especially for plant growth.

## 4. Conclusions

Plants have evolved photoreceptors that respond to different wavelengths of light, with phytochrome playing a crucial role in regulating plant growth and development by absorbing light in red and far-red regions. In LED manufacturing, the combination of Eu^3+^ and Mn^4+^ ions doped into LaAlO_3_ (LAO) can emit efficient red and far-red light suitable for horticulture. This study involved the preparation of LAO ceramic powder samples by a high-temperature solid-state reaction method using metal oxide precursors. The XRD patterns revealed that the dopants have successfully doped into the host's crystal structure homogeneously without major changes in the pattern. SEM images showed that the dopants were uniformly distributed and regularly shaped. The luminescence excitation and emission spectra indicated the effective incorporation of dopants into the host matrix with their respective excitation and emission bands in single doped samples in red and far-red regions due to Eu^3+^ and Mn^4+^ ions, respectively. Even in co-doped sample, Eu^3+^ and Mn^4+^ does not show any efficient energy transfer that quenches their emission intensity. Also a common blue LED could excite them to get an efficient red and far-red emission from LAO:Eu/Mn phosphor material that maintains constant emission color over a period of time. Overall, the results indicate the potential of the synthesized LAO:Eu/Mn phosphor for use in horticulture for red and far-red lighting system.

## Author contributions

S. K. Jakka: methodology, software, validation, formal analysis, data curation, writing – original draft, writing – review & editing, visualization, supervision, M. M. P. Silva: formal analysis, data curation, writing – original draft, M. J. Soares: resources, funding acquisition, K. Pavani: conceptualization, methodology, software, validation, formal analysis, investigation, data curation, writing – original draft, writing – review & editing.

## Conflicts of interest

The authors declare that they have no known competing financial interests or personal relationships that could have appeared to influence the work reported in this paper.

## Supplementary Material
